# Deciphering the Role of Insulin-Like Growth Factor 1 in Endometrial Cancer in Patients With Polycystic Ovary Syndrome: Protocol for a Methodological Approach Using Cell Culture Experiments

**DOI:** 10.2196/48127

**Published:** 2023-11-21

**Authors:** William Atiomo, Fatma Alqutami, Sara Albasha, Mahmood Hachim

**Affiliations:** 1 College of Medicine Mohammed Bin Rashid University of Medicine and Health Sciences Dubai United Arab Emirates

**Keywords:** endometrial cancer, polycystic ovary syndrome, insulin-like growth factor-1, IGF-1, cell culture, United Arab Emirates, PCOS, polycystic ovary syndrome, women's health, gynecology, oncology, cancer, endocrinology, serum, endometrium, endometrial, cell, biology, gene, genetic, genetics, genes

## Abstract

**Background:**

Endometrial cancer (EC) is the most common gynecological cancer in women globally. It is linked to increasing obesity rates and longer life spans. The molecular mechanisms leading to EC are unclear; however, women with polycystic ovary syndrome (PCOS) have a 3- to 5-fold increased EC risk. According to a pilot study conducted in the United Kingdom, insulin-like growth factor-1 (IGF-1) gene and protein were raised in the endometrium and blood of women with EC and PCOS, compared with those without PCOS (controls). Therefore, raised serum IGF-1 levels may contribute to an increased EC risk in women with PCOS, but it is necessary to test this hypothesis since not all studies have demonstrated this association.

**Objective:**

This study aims to investigate the role of IGF-1 in mediating EC risk in PCOS. This will be achieved by evaluating the proliferative effects of PCOS serum, IGF-1, and IGF-1 antagonist on human endometrial cancer 1-A and 1-B cell lines, with a comparison to controls (using serum from women without PCOS and cell culture media). The study will also identify differentially expressed genes and pathways activated by various treatments.

**Methods:**

We intend to recruit 20 women with PCOS and 20 women without PCOS for this cross-sectional study. All experiments will be carried out 4 times to ensure consistency. We will perform transcriptomic and phosphoproteomic profiling to identify differentially expressed genes and phosphoproteins between different treatments using RNA sequencing and phosphoproteomics. We will also perform bioinformatics pathway analysis to identify whether any unique collection of genes or phosphoproteins explains increased EC risk in PCOS. The primary outcome measure will be the cell proliferation (growth) difference measured by cell index values. Our protocol stands out due to its unique approach; no previous study has used this approach to investigate the oncogenic effect of serum from women with PCOS. Additionally, no previous study has considered the differential mutations of genes related to the insulin signaling pathway across various types of human EC cell lines and the potential impact of these variations on their experimental findings.

**Results:**

Participants are currently being recruited. It is expected that preliminary findings suitable for analysis and publication will be available by the summer of 2024.

**Conclusions:**

Although we currently do not have any results to report, sharing our protocol at this stage will aid in research collaboration, provide an opportunity for early feedback, and help reduce duplication of effort by other research groups. The findings of our study will have broader implications. A deeper understanding of the mechanisms underlying the regulation of the IGF system in PCOS and EC will improve our ability to develop effective treatment modalities for EC and will be a vital step toward reducing EC in women globally.

**International Registered Report Identifier (IRRID):**

DERR1-10.2196/48127

## Introduction

### Overview

Endometrial cancer (EC) was the sixth most diagnosed cancer in women globally, in 2020 [1], with 417,000 new cases and 97,000 deaths. The incidence is also increasing rapidly with rising rates of obesity [2] and longer life expectancy. It is estimated that the incidence will increase by more than 50% worldwide by the year 2040 [1]. EC was also the most common gynecological cancer in the United Arab Emirates in 2020, with a 5-year prevalence of 491 per 100,000 individuals [3]. The most common type of EC is type 1 (endometrioid), which is present in 80% of tumors. Treatment often involves hysterectomy—a major surgical operation with serious implications, such as infertility, for younger women [4], which is of growing concern in the United Arab Emirates [5]. There is, therefore, an urgent need for research to prevent and improve the management of EC in the United Arab Emirates and globally. However, uncertainty persists about the precise molecular mechanisms leading to EC, which hinders facilitating EC prevention and treatment.

Some of the known risk factors for EC include polycystic ovary syndrome (PCOS), diabetes, obesity, inflammation, insulin resistance, high estrogen concentrations, and possibly, high circulating insulin-like growth factor 1 (IGF-1) levels [6]. PCOS is, however, the most common female endocrinopathy [7] in women of reproductive age, and potentially, it could account for many cases of EC. PCOS is associated with obesity, infrequent menstrual periods, infertility, hirsutism, enlarged ovaries with many small follicles on ultrasound imaging, increased risks of type-2 diabetes in later life, and a 3- to 5-fold increased risk of EC [8,9], with higher risks in premenopausal women. Mechanisms thought to increase EC risk in PCOS include raised estrogen levels, obesity, anovulatory menstrual cycles, and unopposed (from progesterone) estrogen stimulation of the endometrium; however, the degree to which these mechanisms increase EC risk or interact with each other to underpin EC risk in PCOS is uncertain [10]. Genomic and proteomic approaches [11] have also identified potential new genes and proteins that might explain the link between PCOS and EC and could upregulate genes associated with the insulin signaling pathway in PCOS.

Over the past few years, research studies have investigated the potential role of insulin resistance and IGF-1 in the association between PCOS and EC [12,13]. The possible role of insulin resistance and IGF-1 in the association between PCOS and EC is supported by observations that endometrial dysfunction, causing miscarriages in PCOS, has been linked to insulin resistance and the associated elevation in serum IGF-1 levels in individuals with PCOS [14]. Epidermal growth factor, IGF-1, and IGF-2 were also found to stimulate a time-dependent mitogenic response in human EC cell lines [15]. The most frequently altered mutation in type 1 EC is the phosphoinositide 3-kinase (PI3K) pathway [16]. Elevated IGF-1 and insulin levels have been shown to directly stimulate cell proliferation by activating the phosphatidylinositol-4,5-bisphosphate 3-kinase catalytic subunit alpha pathway [17]. However, the precise association between serum IGF-1 and EC is still uncertain, and results from case-control studies are conflicting [18]. Multiple other molecular pathways also contribute to EC progression. Dysfunction in the TP53 pathway is common in type 2 EC, resulting in the loss of crucial p53 functions. Additionally, the overactivation of the mitogen-activated protein or extracellular signal-regulated kinase (ERK) pathway promotes EC cell proliferation and migration. These pathways, however, interact with IGF-1 signaling, and IGF-1 gene promoter polymorphisms are associated with EC [18].

The importance of investigating the IGF-1 pathway in the association between EC and PCOS is justified. This is because EC is a disease mainly driven by phosphoinositide 3-kinase (PI3K), as the PI3K pathway is aberrant in more than 90% of ECs. PI3K signaling is the main pathway cascade of insulin or IGF-1 signaling [19].

### Prior Work

In a pilot study in which 102 women were recruited, the investigators found for the first time that IGF-1 gene and protein levels were raised in the endometrium and serum of women with PCOS and EC, compared with controls. These two groups exhibited similar levels of obesity [13]. Raised serum IGF-1 levels may, therefore, contribute to an increased risk of EC in women with PCOS; however, there is a need to test this hypothesis since not all studies have demonstrated an association between serum IGF-1, IGF-binding protein 1, IGF-binding protein 3, and insulin levels with EC [20]. A recently published systematic review [21] found that IGF-1 levels in women with PCOS were elevated compared with controls, as found in our pilot study. A total of 20 studies were included in the meta-analysis [21] involving 657 individuals: 362 patients with PCOS and 295 normal controls. The results of the meta-analysis showed that serum IGF-1 levels were significantly higher in patients with PCOS compared to controls (standard mean difference 0.89). Subgroup analysis based on BMI showed that elevated IGF-1 levels were associated with normal-weight and overweight patients in the PCOS group. Further clarification is required about the interplay between PCOS, IGF-1, and the endometrium as well as the impact they have on EC.

### Aims

The aim of this study is to further investigate the possible role of IGF-1 in mediating the risk of EC in women with PCOS.

### Objectives

The objectives of the study are as follows:

- To compare the oncogenic effect of PCOS serum, IGF-1, and IGF-1 antagonist on EC cell lines in women with PCOS and controls (using serum from women without PCOS and cell culture media)
- To identify differentially expressed genes and pathways activated by different treatments used in the previous objective
- Perform a bioinformatics pathway analysis to identify whether any unique genes or nodes can explain the increased EC risk in PCOS

**Figure 1:**
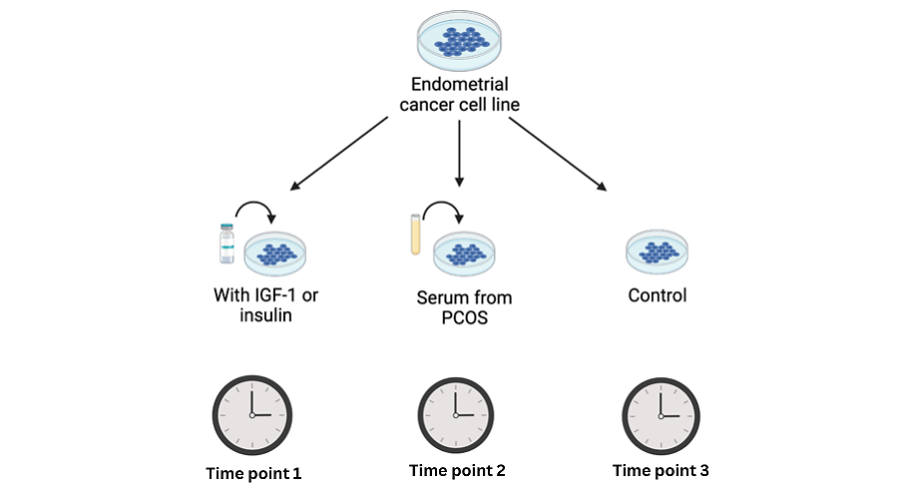
Comparing the oncogenic effect of polycystic ovary syndrome (PCOS) serum on endometrial cancer cell lines with the effect of serum from women without PCOS (controls) and insulin-like growth factor 1 (IGF-1).

Although using cell culture experiments, as described in this paper, is not a novel approach for studying molecular mechanisms to help the prevention and treatment of diseases [22], the unique aspects of our protocol lie in the fact that no previous study has used this approach to investigate the oncogenic effect of serum from women with PCOS. More importantly, no previous study has considered the differential mutations of genes related to the insulin signaling pathway in human endometrial cell lines, as identified in the Cancer Dependency Map Portal [23], and what impact this may have on their experimental findings (Figure 1).

## Methods

### Ethics Approval

Ethics approval for this study has been obtained from the research ethics committees of Mohammed Bin Rashid University of Medicine and Health Sciences (MBRU IRB-2022-149) and Kings College London in Dubai (October 12, 2022). Approvals from other hospital sites (Latifa Women and Children’s Hospital and Mediclinic Hospitals in Dubai) will be sought, as appropriate, based on participant recruitment rates.

### Recruitment

The study will aim to recruit 20 women with PCOS and 20 women without PCOS aged between 18 and 45 years. We will recruit women with PCOS and controls into the study if they have a BMI below 30 and are not on any medications. This is to mitigate the possible confounding effect of obesity on EC risk. Inclusion criteria for women with PCOS will involve a diagnosis using the Rotterdam Criteria [24], which encompasses the presence of 2 or more of the following conditions: clinical or biochemical evidence of hyperandrogenism, chronic oligo-ovulation or anovulation, and the observation of polycystic ovaries on ultrasound, with the exclusion of other causes of chronic oligo-ovulation or anovulation (eg, hyperprolactinemia and hyperthyroidism). Controls will be women with regular menstrual cycles identified from a gynecology or other appropriate outpatient setting at Kings College Hospital, Latifa Women and Children’s Hospital, or Mediclinic Hospital in Dubai, without a diagnosis of polycystic ovaries on ultrasound scan. Male patients will be excluded from this study, as they do not develop EC.

Women identified as having PCOS or controls will be identified by gynecologists in participating hospitals and provided a patient information sheet. If the patient agrees to participate, written consent will be obtained. One of the study investigators will then be informed by the clinician or gynecologist who recruited the patient into the study. The investigator will then contact the participant to arrange a mutually convenient time for sample collection. For women with and without PCOS recruited into the study, participation will not involve any extra visits to the hospital in addition to visits required as part of their clinical care. Fasting blood samples will be obtained following the exclusion of a possible pregnancy. With the permission of the participants, we will review their electronic medical records to obtain details on their medical and gynecological history, including the reason for clinical presentation, menstrual history, date of last menstrual period before sample collection, past medical or surgical history, medication, allergies, and smoking history. The most recent height, weight, and blood pressure measurements in the electronic medical record will be recorded. The details of their last pelvic ultrasound scan will also be recorded to confirm if their ovaries did or did not look polycystic.

Participants can withdraw from the study at any stage without their clinical care being affected. We do not anticipate any issues with adverse effects; however, the research team aims to meet fortnightly as a research group during the study, with steering committee meetings arranged as indicated. The principal investigator will report any relevant data safety issues reported during these meetings or from any other sources to the Mohammed Bin Rashid University’s Institutional Review Board (IRB).

### Blood Tests

Following informed consent, 1 extra sample of blood (in addition to the other blood tests required as part of their clinical care) will be obtained from the participant’s arm and collected into 1 vacutainer for the study. Whole blood collected from patients with PCOS and controls will be stored for a maximum of 24 hours before the serum is separated. Samples will be transported from the gynecology clinic to the laboratory at Mohammed Bin Rashid University, ideally within 6 hours of whole blood collection, in dry ice between 2˚C and 8˚C, with the tubes stored in specimen bags.

We anticipate no major problems with side effects; nonetheless, following the blood tests, the participants may experience minor bruising in the area from which blood was obtained. However, all the blood tests will be taken by an experienced staff member, which should help minimize this risk. Participants will be informed that during the cell culture studies, we may identify incidental factors that might be associated with an increased risk of EC. Should this occur, the results will be made available to participants and their general practitioners or family medicine doctors if they so wish, and they will be offered appropriate counseling. They will also be informed that we may not find any significant differences between blood samples taken from women with PCOS and controls and that there will be a need for further studies on more women to confirm any of our findings. The experiments will not involve any techniques that will put staff at risk or produce environmental contaminants. Participants will also be informed that the potential benefits are that this study might identify new genetic or chemical factors that may prove significant in future studies for the prevention and treatment of EC and that this will be a vital step in reducing the incidence of EC in women living in the United Arab Emirates.

### Processing and Storage of Blood Samples

Whole blood will be allowed to clot for 15-30 minutes at room temperature or left at 4˚C until the reaction is complete. We will aim not to exceed 24 hours and isolate serum within 1 or 2 hours of blood collection. Once the blood has been clotted, it will be centrifuged at 1000 x gravitational units (g) for 10 minutes in a refrigerated centrifuge (at 4˚C) to facilitate the separation of the serum. A barrier will form between the serum and the cells; if no barrier is formed, the sample will be respun for 5 minutes. The serum will be carefully extracted using a fine-bore pipette to prevent the inclusion of red blood cells and will be aseptically transferred to a sterile vial. The samples will be kept at temperatures ranging from 2°C to 8°C during handling. The serum will then be stored without delay at –80˚C in 0.5 mL aliquots to prevent the disintegration of serum proteins. Upon completion of the initial studies with the blood tests obtained from the participants, with their consent, we will retain some of the remaining serum samples for long-term storage to allow further studies if newer technology for carrying out these studies becomes available. However, if participants do not wish to have their serum samples retained for this part of the study, the sample will be discarded in accordance with the established laboratory protocols immediately after the cell culture study. Permission will be sought from the research and ethics committee prior to conducting any further studies using the stored blood samples.

### Objective 1: Comparing the Oncogenic Effect of PCOS Serum on EC Cell Lines With the Effect of Serum From Women Without PCOS (Controls) and IGF-1

**Figure 2:**
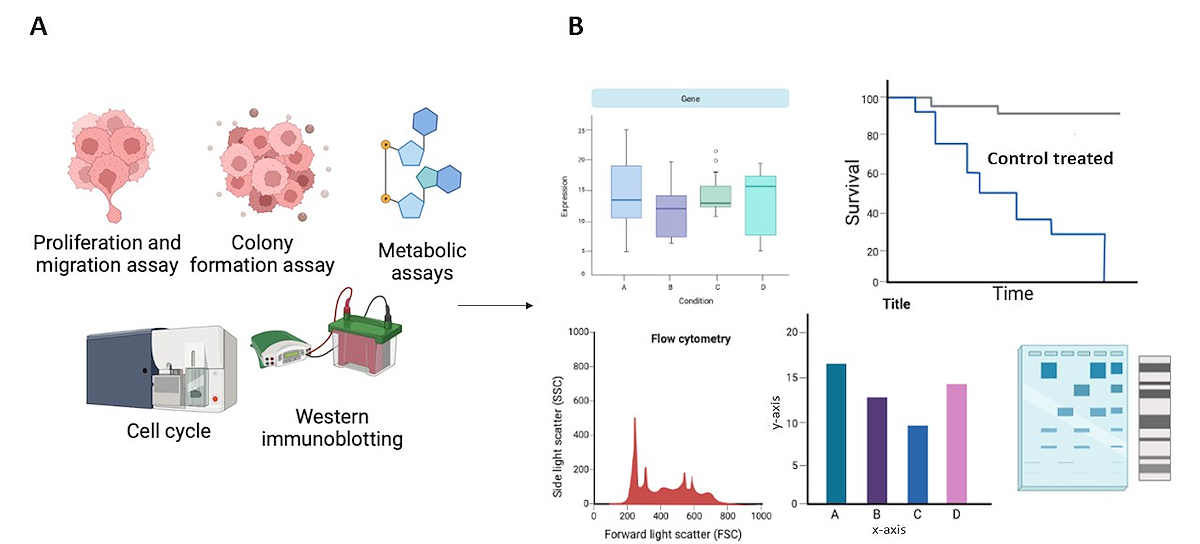
Comparing the oncogenic effect of polycystic ovary syndrome (PCOS) serum on endometrial cancer cell lines with the effect of with the effect of serum from women without PCOS (controls) and insulin-like growth factor 1 (IGF-1). (A) various assays. (B) measures to compare oncogenic effect.

To further investigate the possible role of IGF-1 in mediating the risk of EC in women with PCOS we will evaluate the proliferative effects of PCOS serum, IGF-1, and IGF-1 antagonists on human endometrial cancer 1-A and 1-B cell lines, through a comparison with controls (using serum from women without PCOS and cell culture media; Figure 2).

### Endometrial Carcinoma Cell Lines

Human endometrial cancer (HEC)-1-B and HEC-1-A cell lines will be used in this study. Cells will be treated with the study groups outlined below:

- PCOS serum alone
- PCOS serum and IGF-1
- PCOS serum with IGF-1 antagonist applied
- PCOS serum and IGF-1 with IGF antagonist applied
- Control serum alone
- Control serum and IGF-1
- Control serum with IGF-1 antagonist applied
- Control serum and IGF-1 with IGF antagonist applied
- Cell culture media alone
- Cell culture media and IGF-1
- Cell culture media with IGF-1 antagonist applied
- Cell culture media and IGF-1 with IGF antagonist applied

The treatment will be administered in different time periods, and all experiments will be carried out in quadruplicates. We will examine the effect of cell treatment on cell lines using proliferation, migration, cell cycle analysis, soft agar colony formation assays, and IGF cell signaling. Cell proliferation will be measured using xCELLigence real-time cell proliferation experiment; migration will be measured using xCELLigence real-time cell migration experiment and wound-healing assays; cell cycle analysis will be done using propidium iodide, flow cytometry, and IGF cell signaling, as previously described [25]. Activation of IGF or IGF receptor pathway will be assessed by western blots using the following: antibodies against phospho-IGF-1 receptor, IGF-1 receptor beta-subunit, insulin receptor, protein kinase B (AKT; a serine or threonine protein kinase encoded by the oncogene in the transforming retrovirus isolated from the thymoma cell line AKT-8, derived from the Stock A Strain k AKR mouse originally inbred in the laboratory of Doctor CP Rhoads by KB Rhoads at the Rockefeller Institute), phospho-AKT, phosphor–extracellular signal-regulated kinases (ERKs) 1/2, poly adenosine diphosphate ribose polymerase caspase 3, phosphor–adenosine monophosphate-activated protein kinase, adenosine monophosphate-activated protein kinase, mammalian target of rapamycin and phosphor–mammalian target of rapamycin, as well as PI3K, protein 85 (protein 85 refers to the regulatory subunit of phosphoinositide 3-kinase), ERK1, protein 21, B-cell lymphoma 2, tumor protein 53, phosphatase and tensin homolog, cyclin D1, and caspase 9.

The primary outcome measure will be the difference in cell proliferation measured by cell index values during the xCELLigence real-time cell proliferation experiment. Comparison of cell index values from cells treated in different groups will be performed by the Kruskal-Wallis test followed by multiple comparison tests.

### Objective 2: Identifying Differentially Expressed Genes and Pathways Activated by the Treatment of Endometrial Cell Lines of the Different Study Groups Listed in Objective 1

**Figure 3:**
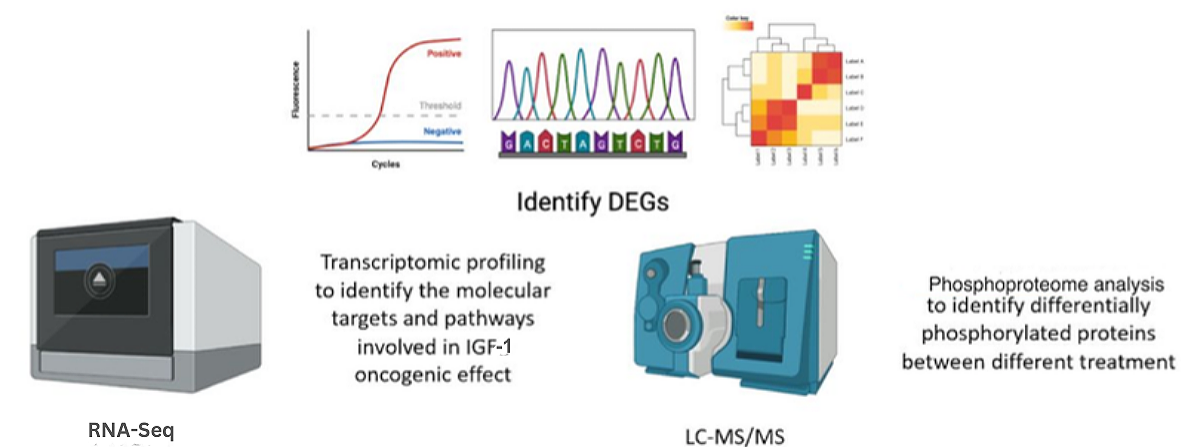
Identifying differentially expressed genes (DEGs) and pathways activated by the treatment in objective 1 of the study protocol. IGF-1: insulin-like growth factor 1. LC-MS/MS: Liquid chromatography with tandem mass spectrometry; RNA-Seq: RNA sequencing.

We will perform transcriptomic profiling of endometrial cell lines to identify differentially expressed genes in response to different study conditions outlined previously, using RNA Sequencing (RNA-Seq) to identify the molecular targets and pathways involved in the oncogenic effects (Figure 3).

The steps for the RNA-Seq include the following:

- Total RNA will be extracted using a RNeasy extraction kit, with on-column DNase digestion.
- RNA quality will be assessed using an Agilent bioanalyzer.
- Samples will be analyzed on an Illumina HiSeq platform using standard protocols.
- For quantitative reverse transcriptase PCR validation of RNA-Seq results, a subset of the groups described in objective 1 will be used.
- The expression of identified differentially expressed genes will be compared in the publicly available RNA sequencing databases.

At the same time, we will use phosphoproteome analysis to identify differentially phosphorylated proteins between different study conditions using liquid chromatography with tandem mass spectrometry measurement.

The steps involved in the phosphoproteomics will be as outlined below:

- Cell lysis, protein digestion, and labelling
- Peptide enrichment, immunoprecipitation, and offline fractionation
- Mass spectrometry data acquisition
- Peptide and protein database searching
- Proteomics data analysis
- Phosphoproteomics and acetyl-immunoprecipitation data analysis

### Objective 3: Bioinformatics

**Figure 4:**
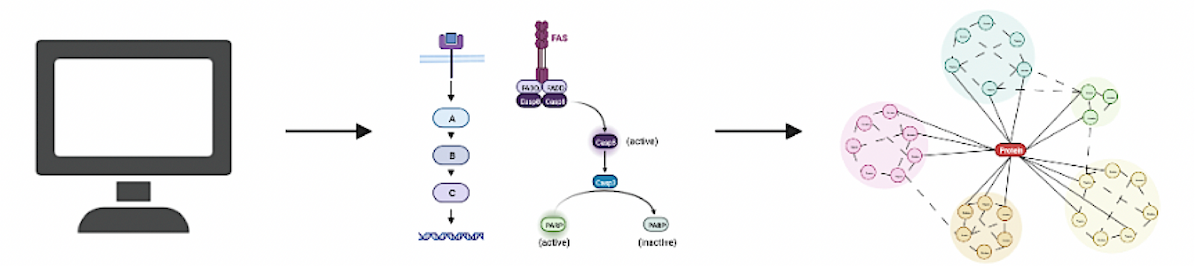
Bioinformatics pathway analysis to identify any key genes and proteins involved in the association between polycystic ovary syndrome (PCOS) and endometrial cancer (EC).

We will perform bioinformatics pathway analysis to identify any key genes and proteins involved in the association between PCOS and EC, after transcriptomic and phosphoproteomics profiling, as identified in objective 2 (Figure 4). The results from the same HEC-1-B and HEC-1-A cell lines and treatment groups, as outlined in objectives 1 and 2, will be used.

The methodology for the RNA-Seq data analysis will be as previously published [26], paired end raw reads (FASTQ format) will be quality- and adapter-filtered using Trim Galore wrapper for FastQC and Cutadapt [27]. The retained paired reads will be aligned to the Ensembl-annotated HG19 human Illumina iGenome build using Tophat2. Subsequently, differential gene expression will be calculated for the different cell culture study groups in comparison to the control specimen, using Cuffdiff, based on fold changes >1.5 and a *P* value <.05. Statistically significantly enriched gene ontologies and pathways for differentially expressed genes will be obtained using WebGestalt and the Cytoscape GeneMANIA plug-in. Next-generation RNA-Seq and associated clinical information will be obtained from publicly available data sets.

The methodology for the phosphoproteomics data analysis will also be as previously published data analysis for the phosphoproteomics data; label-free quantitative data will be filtered with a cutoff of ≥2 observations in the 4 repeats of at least 1 time point. Log transformation will be performed before any missing data points are imputed using random values generated from a normal distribution centered at the 1% quantile and the median SD. The abundance of phosphopeptides will be normalized to the abundance of corresponding parental proteins. The Limma package will be used in the R environment to identify differential proteins or phosphorylation with a false discovery rate–corrected ANOVA *P* value of .05. The k-means clustering approach will be used to analyze the patterns of the differential proteins and phosphorylation events. For functional annotation and network analysis, the differentially regulated proteins will be searched against the String database, and the networks will be visualized by using Cytoscape.

Although the study will aim to recruit 20 women with PCOS and 20 women without PCOS aged between 18 and 45 years, we will begin with a pilot study of samples from 3 women with PCOS and 2 controls and perform validation studies in the larger cohort of 20 women with PCOS and 20 women. The experimental plate design for the cell-culture studies in this feasibility study is illustrated in Table 1.

It will be 1 plate per cell line for each time point (24 h and 48 h). All experiments will be conducted in quadruplicates. We recognize that opinions differ about the ideal sample size for cell-culture experiments [28]; however, we believe our proposed experiments are stringent enough to make the results believable.

**Table 1:** Experimental plate design for cell culture studies.

Plates											
1	2	3	4	5	6	7	8	9	10	11	12
PCOS^a^ 1	PCOS 1 + IGF^b^	PCOS 1 + Inhibitor	PCOS 1 + IGF + Inhibitor	PCOS 2	PCOS 2 + IGF	PCOS 2 + Inhibitor	PCOS 2 + IGF + Inhibitor	PCOS 3	PCOS 3 + IGF	PCOS 3 + Inhibitor	PCOS 3 + IGF + Inhibitor
PCOS 1	PCOS 1 + IGF	PCOS 1 + Inhibitor	PCOS 1 + IGF + Inhibitor	PCOS 2	PCOS 2 + IGF	PCOS 2 + Inhibitor	PCOS 2 + IGF + Inhibitor	PCOS 3	PCOS 3 + IGF	PCOS 3 + Inhibitor	PCOS 3 + IGF + Inhibitor
PCOS 1	PCOS 1 + IGF	PCOS 1 + Inhibitor	PCOS 1 + IGF + Inhibitor	PCOS 2	PCOS 2 + IGF	PCOS 2 + Inhibitor	PCOS 2 + IGF + Inhibitor	PCOS 3	PCOS 3 + IGF	PCOS 3 + Inhibitor	PCOS 3 + IGF + Inhibitor
PCOS 1	PCOS 1 + IGF	PCOS 1 + Inhibitor	PCOS 1 + IGF + Inhibitor	PCOS 2	PCOS 2 + IGF	PCOS 2 + Inhibitor	PCOS 2 + IGF + Inhibitor	PCOS 3	PCOS 3 + IGF	PCOS 3 + Inhibitor	PCOS 3 + IGF + Inhibitor
Control 1	Control 1 + IGF	Control 1 + Inhibitor	Control 1 + IGF + Inhibitor	Control 2	Control 2 + IGF	Control 2 + Inhibitor	Control 2 + IGF + Inhibitor	Media (FBS^c^)	Media (FBS) + IGF	Media (FBS) + Inhibitor	Media (FBS) + IGF + Inhibitor
Control 1	Control 1 + IGF	Control 1 + Inhibitor	Control 1 + IGF + Inhibitor	Control 2	Control 2 + IGF	Control 2 + Inhibitor	Control 2 + IGF + Inhibitor	Media (FBS)	Media (FBS) + IGF	Media (FBS) + Inhibitor	Media (FBS) + IGF + Inhibitor
Control 1	Control 1 + IGF	Control 1 + Inhibitor	Control 1 + IGF + Inhibitor	Control 2	Control 2 + IGF	Control 2 + Inhibitor	Control 2 + IGF + Inhibitor	Media (FBS)	Media (FBS) + IGF	Media (FBS) + Inhibitor	Media (FBS) + IGF + Inhibitor
Control 1	Control 1 + IGF	Control 1 + Inhibitor	Control 1 + IGF + Inhibitor	Control 2	Control 2 + IGF	Control 2 + Inhibitor	Control 2 + IGF + Inhibitor	Media (FBS)	Media (FBS) + IGF	Media (FBS) + Inhibitor	Media (FBS) + IGF + Inhibitor

^a^PCOS: polycystic ovary syndrome.

^b^IGF: insulin-like growth.

^c^FBS: fetal bovine serum.

## Results

In this study, we have described the protocol for an ongoing study to investigate the role of IGF-1 in mediating the risk of EC in women with PCOS. The study will aim to recruit 20 women with PCOS and 20 controls and measure the effect of serum from these women on cell proliferation and molecular pathways in human EC cell lines, compared with IGF-1 and IGF-1 antagonists. Recruitment for the study is currently ongoing. The project was funded in June 2022 and approved by the Mohammed Bin Rashid University of Medicine and Health Sciences’ IRB in October 2022; funds were released in January 2023, and data collection began on 16 March 2023 and is ongoing. Three women with PCOS and 2 controls have been recruited as the date of this submission from 1 hospital site and IRB permission for further participant recruitment at a second hospital site was granted in October 2023. Data analysis has not yet commenced. We anticipate that initial results for analysis and publication will be available by the summer of 2024.

## Discussion

### Expected Outcomes

Although the protocol (using cell culture) described in this paper is not novel for studying molecular mechanisms to help in the prevention and treatment of diseases, the unique aspect of it is that no previous study has used this approach to investigate the oncogenic effect of serum from women with PCOS. More importantly, no previous study has considered the differential mutations of genes related to the insulin signaling pathway in human endometrial cell lines and the potential impact this may have on their experimental findings.

A possible limitation of our protocol includes the risk that the IGF-1 present in the serum of women with PCOS may become diluted in the cell culture media to an extent where the in vitro effects do not represent the in vivo activity. We will, however, aim to mitigate this risk by using Sep-Pak C18 for the extraction, concentration, and fractionation of peptides from PCOS and control serums [29]. We will also begin with a pilot study of samples from 3 women with PCOS and 2 controls to identify any other potential methodological problems before performing validation studies in the larger cohort of 20 women with PCOS and 20 women without PCOS. Another limitation of our protocol is that the use of these cell lines may limit the generalizability of the findings from our study. However, our results may inform future studies by independent research groups using primary cell lines or patient tumor samples.

We are aware of only 1 study [30] in which serum from women with PCOS has been used in experiments on EC cell lines. The study, however, was not designed to answer our research question, as it investigated the potential stimulatory effects of serum exosomes isolated from patients with PCOS on EC cell lines and explored the underlying mechanism. The study [30] found that EC cell lines exposed to exosomes derived from the serum of patients with PCOS exhibited an enhanced migration and invasion phenotype. The study also found that levels of 55 mature micro RNAs significantly differed in serum exosomes from patients with PCOS compared to those from normal controls and that MiR-27a-5p was the most significantly elevated in the serum exosomes of patients with PCOS. Furthermore, in vitro experiment results confirmed that MiR-27a-5p prohibited migration and invasion via Suppressor of Mothers Against Decapentaplegic-4 downregulation, suggesting that serum exosomal MiR-27a-5p may play a role in EC development in patients with PCOS. The implications of these findings for our study are that we may also find enhanced migration and invasion in our PCOS group compared to controls, independent of whether IGF-1 antagonist is present, which would imply that pathways other than IGF-1 may also be at play in the association between PCOS and EC. However, the planned transcriptomic profiling in our study will offer the opportunity to identify any interactions between messenger RNAs and IGF-1 in the possible association between PCOS and EC.

### Conclusions

In conclusion, this study describes a protocol for an ongoing study to investigate the role of IGF-1 in mediating the risk of EC in women with PCOS, by investigating the effect of serum from women with PCOS and controls on cell proliferation and molecular pathways in human EC cell lines. Although we currently do not have any results to report, reporting our protocol should aid research collaboration and early feedback; it could also reduce duplication of effort by other research groups. The broader implications of the findings of our study, when available, are that a deeper understanding of the mechanisms underlying the regulation of the IGF system in PCOS and EC will improve our ability to develop effective treatment modalities for EC. Specifically, investigating how IGF-1 in PCOS participates in the development of EC may identify novel genetic associations between EC and PCOS. This will also facilitate future systems biology–based pathway analysis to explore further molecular mechanisms leading to EC and will be a vital step in reducing EC in women globally.

## Abbreviations

AKT: protein kinase B
EC: endometrial cancer
ERK: extracellular signal-regulated kinase
HEC: human endometrial cancer
IGF-1: insulin-like growth factor 1
IRB: Institutional Review Board
PCOS: polycystic ovary syndrome
PI3K: phosphoinositide 3-kinase
RNA-Seq: RNA sequencing

## Data Availability

Data sharing is not applicable to this study, as no new data were created or analyzed.
